# Lupus and the Lungs: The Assessment and Management of Pulmonary Manifestations of Systemic Lupus Erythematosus

**DOI:** 10.3389/fmed.2020.610257

**Published:** 2021-01-18

**Authors:** Raj Amarnani, Su-Ann Yeoh, Emma K. Denneny, Chris Wincup

**Affiliations:** ^1^Department of Rheumatology, University College London Hospital, London, United Kingdom; ^2^Division of Medicine, Department of Rheumatology, University College London, London, United Kingdom; ^3^Department of Respiratory Medicine, University College London Hospital, London, United Kingdom; ^4^Leukocyte Trafficking Laboratory, Centre for Inflammation and Tissue Repair, UCL Respiratory, University College London, London, United Kingdom

**Keywords:** systemic lupus erythematosus (SLE), interstitial lung disease (ILD), pleurisy, pleural effusion, shrinking lung syndrome, pulmonary arterial hypertension, acute lupus pneumonitis, pulmonary vasculitis

## Abstract

Pulmonary manifestations of systemic lupus erythematosus (SLE) are wide-ranging and debilitating in nature. Previous studies suggest that anywhere between 20 and 90% of patients with SLE will be troubled by some form of respiratory involvement throughout the course of their disease. This can include disorders of the lung parenchyma (such as interstitial lung disease and acute pneumonitis), pleura (resulting in pleurisy and pleural effusion), and pulmonary vasculature [including pulmonary arterial hypertension (PAH), pulmonary embolic disease, and pulmonary vasculitis], whilst shrinking lung syndrome is a rare complication of the disease. Furthermore, the risks of respiratory infection (which often mimic acute pulmonary manifestations of SLE) are increased by the immunosuppressive treatment that is routinely used in the management of lupus. Although these conditions commonly present with a combination of dyspnea, cough and chest pain, it is important to consider that some patients may be asymptomatic with the only suggestion of the respiratory disorder being found incidentally on thoracic imaging or pulmonary function tests. Treatment decisions are often based upon evidence from case reports or small cases series given the paucity of clinical trial data specifically focused on pulmonary manifestations of SLE. Many therapeutic options are often initiated based on studies in severe manifestations of SLE affecting other organ systems or from experience drawn from the use of these therapeutics in the pulmonary manifestations of other systemic autoimmune rheumatic diseases. In this review, we describe the key features of the pulmonary manifestations of SLE and approaches to investigation and management in clinical practice.

## Introduction

Systemic lupus erythematosus (SLE) is a chronic, autoimmune disorder that can present with a wide array of clinical and immunological abnormalities (1). Pulmonary manifestations of the disease include disorders of the lung parenchyma, pleura, and pulmonary vasculature. Furthermore, some SLE therapies predispose to an increased risk of respiratory infections (2).

Clinical assessment of patients with SLE should routinely consider careful evaluation for respiratory involvement. Symptoms including dyspnea, pleuritic chest pain, reduced exercise tolerance, cough, and hemoptysis should prompt investigation for potential underlying lung disease (3, 4). However, it is important to consider that some asymptomatic patients may also present with incidental findings of abnormal chest imaging or lung function tests in the absence of overt respiratory symptoms (5). It is also important to consider whether these symptoms are occurring in the context of active SLE involving other organ systems. Serological evidence of increased disease activity including elevated erythrocyte sedimentation rate (ESR), low complement, and increased double-stranded DNA (dsDNA) antibody titers should also prompt the clinician to consider whether new respiratory symptoms are directly attributed to lupus.

The exact prevalence of SLE-related lung disease is unknown and previous studies have varied widely in their estimates. Most report that between 20 and 90% of SLE patients will experience some form of lung involvement during the course of their disease (6, 7). However, more recently it has been suggested that this figure lies between the range of 50–70% (8). Predictors for progression to earlier permanent lung damage, include older age and those positive for anti-RNP antibodies (9). Pulmonary manifestations of SLE are associated with a higher mortality rate (10) and this varies depending upon the exact type and extent of lung involvement seen. More chronic forms of lung disease relating to SLE can have a significant negative affect on patient wellbeing, physical performance status, and are detrimental to quality of life (11).

In this review, we discuss the latest understanding on the ways in which lupus can affect the respiratory system, highlight how these patients may present clinically, and outline current approaches for investigation and management.

## Diseases of the Lung Parenchyma

### Interstitial Lung Disease (ILD)

The estimated prevalence of SLE-associated interstitial lung diseases (ILD) is suggested to be between 3 and 9% (12, 13). Although ILD is highly prevalent in rheumatoid arthritis and other systemic autoimmune rheumatic diseases (such as scleroderma and anti-synthetase syndrome), it is relatively uncommon in SLE (8). A small study previously reported that clinical progression of ILD in SLE is slow and often stabilizes over time (12). Risk factors for developing SLE-associated ILD include longstanding disease, older age and overlapping clinical features with scleroderma such as Raynaud's phenomenon and sclerodactyly (14–17). Various forms of ILD have been described in SLE including non-specific interstitial pneumonia (NSIP), organizing pneumonia, lymphocytic interstitial pneumonia, follicular bronchitis, and usual interstitial pneumonia (18–21). Bronchiolitis obliterans has also been reported as an initial manifestation of SLE (19).

Patients present similarly in most types of ILD with symptoms such as cough and dyspnea although it is important to consider that some may be asymptomatic (22). Diagnosis of SLE-associated ILD can be made with high resolution computed tomography (HRCT) and excluding other potential causes of ILD (such as screening for overlap disorders by measuring rheumatoid factor, serum muscle enzymes, an extended myositis panel and anti-centromere autoantibodies) (23). Checking extractable nuclear antigens (ENA) should also be considered as previous studies have demonstrated that patients with anti-La, anti-Scl-70 and anti-U1RNP antibodies were more likely to develop ILD. Interestingly, anti-dsDNA antibody titer do no associate with the development of ILD (24). Lung function tests may show a restrictive pattern of disease and a decrease in diffusing capacity for carbon monoxide (DLCO) (8). Histological studies have reported the presence of lymphocytic and mononuclear interstitial and peribronchiolar infiltrates in biopsies taken from those with SLE-related NSIP (25).

There are a lack of clinical trials assessing the treatment of SLE-related ILD and in particular there are no head-to-head studies. Therefore, recommendations are predominantly based on case reports, small case series, physician expertise, and by applying findings from studies of ILD in other autoimmune rheumatic diseases. Intravenous cyclophosphamide was reported to show significant improvement vital capacity in two SLE patients with ILD in which both patients presented with pleuritic chest pain in the context of active SLE (26). Another case report noted that oral methotrexate resulted in a marked improvement in lung function in a patient with SLE-related ILD (27). An observational study of 14 patients with SLE-associated ILD reported that three patients showed significant improvement with high dose oral steroids (60 mg prednisolone daily for a minimum of 4 weeks). Six of the 14 patients had an improvement in respiratory symptoms and all were treated with systemic steroids (18). Three patients within the cohort died, two of pulmonary fibrosis, and one from infection thus highlighting the clinical challenge posed by immunosuppressive therapy in the context of SLE-related ILD. It is important to consider that this study was published in 1990 and thus predates a number of the newer treatments available for the management of SLE, such as mycophenolate mofetil (MMF), rituximab and belimumab.

Current treatment often includes the use of high dose corticosteroids along with agents such as cyclophosphamide and rituximab in severe cases (28, 29) to induce remission. Steroid-sparing agents such as MMF and azathioprine may be used in milder cases or in maintaining long-term control of the disease (30, 31).

### Acute Lupus Pneumonitis

In some cases, chronic ILD may be the long-term sequelae of an acute process, for example acute lupus pneumonitis. This is a rare manifestation of SLE that has been reported to occur in 1–4% of patients (32). Clinically, acute lupus pneumonitis presents in the context of a systemic flare of SLE in addition to dyspnea, cough (including hemoptysis) and pleuritic chest pain. Fever is commonly associated with the acute presentation, thus making it a clinical challenge to differentiate from infection. There is limited data on lung histology in acute lupus pneumonitis, although reports of lymphocytic infiltrates and alveolar damage with associated interstitial edema have been reported in both lung biopsy samples and at post-mortem assessment (24).

Acute lupus pneumonitis may also be the initial presenting symptom of SLE. A case series of five patients in which acute lupus pneumonitis was the first feature of SLE reported that all five were female, aged 14–26 years old. They were all ANA positive, whilst three were also positive for anti-dsDNA antibodies. Fever was present in all cases with cough as a presenting symptom in four of the five patients, with hypoxia noted in three. All patients received corticosteroids and four patients were treated with cyclophosphamide either as monotherapy or in combination with intravenous immunoglobulins (IVIg). The one patient who did not receive cyclophosphamide was treated with azathioprine. Three patients survived but two died as a result of infection (33). Others have also reported the use of IVIg in acute lupus pneumonitis (34, 35). Given that the differential diagnosis in this presentation often includes bacterial pneumonia, and as infection can commonly co-exist with acute lupus pneumonitis, IVIg represents a useful option as it does not convey the high risk of immunosuppression associated with other agents. It is also important to consider using broad spectrum antibiotics (in particular directed against encapsulated organisms) if there are concerns about intercurrent infection. Further, prompt initiation of systemic glucocorticoid therapy has been reported to be of benefit in reducing mortality rates. Additional treatments that have been used in the management of acute lupus pneumonitis are similar to those used in SLE-related ILD, such as high dose glucocorticoids in combination with either MMF, azathioprine, rituximab, or cyclophosphamide. However, in spite of this the outcomes are often poor with associated high mortality rates (33, 36).

## Pleural Disease

Pleural involvement is the most common SLE-related lung disease (37). Clinically, patients often present with pleuritic chest pain, cough and dyspnea due to inflammation of the pleura (38). Patients may have an associated pleural effusion which is often bilateral and exudative in nature (39, 40). Estimates suggest that between 30 and 50% of SLE patients will develop a pleural effusion at some point during their disease course, although often these are small and may not result in obvious symptoms (39, 41).

Diagnosis of pleural involvement in SLE is usually clinical with typical features in the patient history. It is however important to exclude other causes of pleural inflammation that can occur in SLE including infection, pulmonary embolism, malignancy, congestive cardiac failure (37), or pericarditis, which may present in a similar manner. Drug-induced pleuritis from agents such as hydralazine, procainamide and anti-tumor necrosis factor-alpha medications should also be considered (42–44). In such cases, drug cessation is often sufficient to resolve symptoms.

Although not necessary for diagnosis, if there is clinical uncertainty as to the cause of a pleural effusion, aspiration can be performed. Pleural fluid in patients with SLE classically show elevated levels of protein, lactate dehydrogenase (LDH), leukocytes, and in some cases ANA positivity (37, 39).

The mainstay treatment of pleurisy in SLE has traditionally been non-steroidal anti-inflammatory drugs (NSAIDs) with some patients requiring corticosteroids (38). Rarely, other steroid-sparing agents such as azathioprine, methotrexate, cyclosporine, and cyclophosphamide may be indicated (37). In refractory disease, there have been cases showing effective use of pleurodesis (45, 46).

## Disorders of the Pulmonary Vasculature

### Pulmonary Arterial Hypertension (PAH)

Pulmonary arterial hypertension (PAH) is a progressive disorder characterized by a resting mean pulmonary artery pressure above 25 mmHg and a pulmonary wedge pressure below 15 mmHg (47). There are a number of possible underlying causes that may result in PAH in SLE, including left ventricular dysfunction or congestive cardiac failure that may be a result of the increased risk of atherosclerosis associated with SLE. It may also be a manifestation of the long-term sequelae of parenchymal lung diseases (such as ILD) or chronic thromboembolic disease (48). Studies estimate the prevalence of PAH in SLE to be in the range of 1–43% depending on the cohort (49–54). A recent comprehensive meta-analysis assessing the prevalence of PAH found an estimated pooled prevalence of 8% (55). Despite this, severe PAH is thought to be a rare manifestation in SLE and is not included in the Systemic Lupus Erythematosus Disease Activity Index 2000 (SLEDAI-2K) disease activity score (56).

Clinical symptoms of PAH in SLE are often non-specific and range from generalized fatigue and weakness to chest pain and dyspnea at rest (48). Initial investigations often include an electrocardiogram that may show right ventricular hypertrophy and right axis deviation. Radiographic imaging with computerized tomography may be used to exclude other diseases such as ILD and will often show enlarged pulmonary vessels (57). Echocardiography can estimate systolic pulmonary artery pressure and is therefore a vital non-invasive tool to assist in making a diagnosis. However, even with a suggestive echocardiogram result and high clinical suspicion, right heart catheterization remains the “gold standard” test to confirm the diagnosis (58).

Management of PAH in SLE is similar to that of idiopathic PAH. However, most randomized controlled trials that have specifically analyzed the management of PAH associated with connective tissue diseases often have not included a subgroup analysis of SLE patients (48). Drugs such as phosphodiesterase-5 inhibitors, endothelin receptor antagonists and prostacyclin pathway agonists have all shown to be effective in SLE associated PAH to varying degrees (59–64). More recently, the guanylate cyclase stimulator riociguat has shown to be effective in a small number of SLE-associated PAH cases (65, 66).

Numerous observational cohort studies have also noted benefit with corticosteroids and immunosuppressive therapy including cyclophosphamide, cyclosporine and MMF (67–70). One case report has also described effective use of rituximab in refractory SLE-associated PAH (71). Overall, it is generally thought that a combination of both immunosuppression and traditional PAH treatment should be used together to enhance long-term outcomes (68).

### Pulmonary Embolic Disease

Pulmonary embolism (PE) also needs to be considered in the acute setting in any patient with SLE who presents with pleuritic chest pain (especially if associated with acute hypoxia). In the more chronic setting, chronic pulmonary embolic disease can also lead to pulmonary hypertension (chronic thromboembolic pulmonary hypertension). It is particularly important to consider embolic disease in those patients who have secondary anti-phospholipid syndrome (APS), given the obvious increased risk of thrombosis associated with the disease. Previous studies have reported that one-third of patients with SLE will have positive anti-phospholipid antibodies and those with a positive lupus anticoagulant have previously been shown to have a six-fold increased risk of venous thrombosis. In comparison, a positive anti-cardiolipin antibody carried twice the risk when compared with SLE patients without positive anti-cardiolipin antibodies (72). Previous studies have also reported that patients with SLE, even in the absence of APS, are at an increased risk of unprovoked PE when compared with the general population and therefore the absence of positive anti-phospholipid serology should not be falsely reassuring.

The “gold standard” investigation for PE is computed topography pulmonary angiogram (CTPA), which can identify the presence of thrombosis within the pulmonary vasculature. However, it is important to consider that SLE patients presenting with pleuritic chest pain and hypoxia may instead be suffering from pleurisy (as described above). Results from the Michigan Lupus Cohort assessed the outcomes of 182 patients with SLE who had previously undergone a total of 357 CTPA scans. The authors found a significant decrease in the likelihood of confirming PE in patients who had previously had three or more scans, thus suggesting that repeated scanning of patients without a previously proven PE is unlikely to confirm a new diagnosis (73).

In the context of PE associated with APS, lifelong anticoagulation is likely to be recommended. Recent studies investigating direct oral anticoagulants have recommended against their use in arterial thrombosis, such as PE (74).

### Pulmonary Vasculitis and Pulmonary Hemorrhage

Pulmonary vasculitis, or diffuse alveolar hemorrhage (DAH), is a rare but severe manifestation of SLE that is associated with a high mortality rate of up to 90% (75). This has been reported to affect <5% of patients with SLE and is more commonly seen concurrently in the context of active lupus nephritis (76). In addition, this manifestation has been reported to be the initial presentation of SLE in ~20% of all cases, which means that it is important to consider lupus in any new case of pulmonary hemorrhage in which an alternate underlying cause is not present (77). It has also been reported that patients with secondary APS may be at increased risk of DAH and that this may also occur *de novo* in patients with SLE who are have anti-phospholipid antibodies without previous thrombotic events. This suggests that this is not entirely the result of anticoagulant therapy and may represent an as yet unclassified mechanism for pulmonary vasculitis (78). As with other acute pulmonary manifestations of SLE, the symptoms can often mimic infection thus making the diagnosis a challenge.

Findings from small cases series and cohort studies have highlighted that dyspnea and pulmonary infiltrates on thoracic imaging are almost universally in seen. Fever is reported in the majority of cases although occult hemoptysis is only seen in just over half of patients at presentation (79). Many patients will also present with extrapulmonary manifestations of SLE to suggest a generalized systemic flare of the disease. More subtle signs that suggest DAH include pleural effusions and anemia is seen in nearly all cases, and may be present before signs such as hemoptysis are observed (75, 80). Imaging studies often describe classical bilateral alveolar interstitial infiltrates. Many patients are deemed clinically unstable for further dedicated investigation however those that proceed to bronchoscopy are usually found to have high neutrophil count, low lymphocyte count and hemosiderin-laden macrophages within the lavage and occult blood often seen (79, 81). If the patient is able to tolerate pulmonary function tests then an elevated DLCO is usually indicative of alveolar hemorrhage.

Given a lack of clinical trial data from DAH in SLE, treatment recommendations are usually based upon other autoimmune conditions associated with pulmonary hemorrhage (such as ANCA-associated vasculitis) and often include pulsed intravenous steroids in combination with cyclophosphamide (79), rituximab, plasmapheresis, and IVIg (81, 82).

## Shrinking Lung Syndrome (SLS)

Shrinking lung syndrome (SLS) is an uncommon manifestation of SLE with an estimated prevalence of ~1–2% (9, 83, 84). The exact cause of SLS is unclear, however it is believed to involve abnormal diaphragmatic strength and may be related to due to impaired phrenic nerve signaling (85).

Patients with SLS often present with symptoms of pleuritic chest pain and progressive dyspnea (86). Due to its rarity, there is no diagnostic criteria for SLS. Lung function tests often show a restrictive defect with a reduction in lung volume and DLCO (84). Radiographic imaging in SLS is often non-specific with occasional elevation of the diaphragm and basal atelectasis with usually no evidence of interstitial lung or pleural disease (87). It is also important to consider other conditions before a diagnosis of SLS is made including central nervous system disorders and diaphragmatic palsies (88).

Evidence for the optimal management of SLS is limited. Corticosteroids and immunosuppressive agents including azathioprine, MMF and rituximab have been used to varying degrees of efficacy (86, 89–92). Some have suggested the use of hematopoietic cell transplantation (93) and beta agonist therapy (94) in SLS. Others have reported some benefit in the use of theophylline thought to be helpful by improving diaphragmatic strength (87, 95). Comprehensive studies have generally shown a good prognosis with treatment in most SLS patients (87, 88).

## Conclusions

Pulmonary manifestations of SLE can present with a wide array of symptoms and can often be difficult to differentiate from other conditions, most notably infection. The key differences between these disorders are summarized in Table 1.

**Table 1:** A summary of the way in which pulmonary manifestations of systemic lupus erythematosus (SLE) may present in clinical practice, the underlying pathogenesis and relevant treatment options.

**Diagnosis**	**Presentation**	**Pathogenesis**	**Relevant investigation findings**	**Histological features**	**Treatment**
Interstitial lung disease	Chronic, often progressive dyspnea Cough (often non-productive) Possible evidence of scleroderma, anti-synthetase syndrome, or rheumatoid arthritis May be asymptomatic	Poorly understood/unclear Likely a result of the aberrant inflammatory response due to imbalance of pro- and anti-inflammatory cytokine release (96) Possibly the result of repeated alveolar injury resulting in a combination of both impaired apoptosis and abnormal fibroblast proliferation	Infiltrative changes on CXR or HRCT chest Restrictive pattern on pulmonary function tests with reduced DLCO Test for auto-antibodies suggestive of overlap disorder (e.g., RhF, anti-CCP, anti-centromere, anti-Scl-70, anti-RNP) and muscle enzymes (CK, LDH)	Mononuclear or lymphoplasmacytic interstitial and peribronchiolar infiltrates (particularly in NSIP pattern disease) Interstitial fibrosis present. Deposits of IgG, IgM, C1q, and C3 within alveolar septae previously reported (14)	Depends upon severity *Severe or rapidly progressive* Oral/IV corticosteroids followed by either Cyclophosphamide, Rituximab, MMF, Azathioprine
Acute lupus pneumonitis	Acute dyspnea Fever Cough (usually non-productive but occasional hemoptysis) Features of extrapulmonary SLE disease activity	Rapid systemic inflammatory response resulting in acute damage to the lung parenchyma. Alveolar injury resulting from direct immune-mediated inflammation	CXR – diffuse bilateral alveolar infiltrates CT thorax – previous reports of ground-glass changes Serological evidence of lupus activity (low complement and elevated anti-dsDNA antibody titers)	Often non-specific Features can include alveolar wall damage, necrosis, inflammatory infiltrate, oedema, hemorrhage, hyaline membranes (97) Capillary microangiitis, fibrin thrombi and necrotic neutrophils have also been described (98)	Systemic corticosteroids (either high dose oral or pulsed IV) plus either Cyclophosphamide, Rituximab, MMF, Azathioprine Possibly IVIg
Pleurisy	Chest pain (often pleuritic in nature) Cough Dyspnea Physical signs such as pleural rub may be present	Inflammatory infiltration into the pleura	Raised CRP Imaging usually normal CXR ± CT thorax or CTPA helpful to rule out other causes	Non-specific inflammatory changes associated with fibrin deposition along with pleural fibrosis (99)	*Mild* Oral NSAIDs *Moderate* Oral corticosteroids *Severe (rarely required)* IV corticosteroids, Azathioprine, Cyclophosphamide, Rituximab, MMF
Pleural effusion	Dyspnea Chest pain, usually associated with pleurisy May be asymptomatic Physical signs including reduce basal air entry and decreased resonance	*As per “Pleurisy”* Excessive inflammation results in exudative fluid secretion between pleural lining resulting in effusion	Effusion(s), usually bilateral, present on CXR or CT thorax Aspirate (if underlying diagnosis in doubt) – elevated protein, LDH, leukocytes, ANA positive in some cases	Predominantly based on cytological features Pleural fluid may show characteristic lupus erythematosus (LE) cells, e.g., neutrophils or macrophages containing intracellular evidence of phagocytosed lymphocyte nuclei (100)	Corticosteroids Drainage if large Pleurodesis in recurrent or refractory cases Cessation of any potential drug causes
Pulmonary arterial hypertension	Can be non-specific (such as fatigue and weakness) Progressive dyspnea Occasional chest pain Physical signs may show right ventricular heave	*Dependent upon underlying cause* Left ventricular dysfunction/congestive cardiac failure may result from direct myocardial inflammation from SLE (e.g., myocarditis) or as a result of enhanced atherosclerosis Chronic thromboembolic disease may result from pro-coagulant factors such as aPl antibodies Lung parenchymal disease as the result of direct inflammatory response in lung tissue Dysregulation between vasoconstrictive and vasodilatory mediators	EKG – RVH and right axis deviation Echocardiogram – elevated PASP, TR Right heart catheterization – mean arterial pressure ≥25 mm Hg confirms diagnosis CT thorax – useful to exclude other secondary causes CTPA – useful to rule out chronic embolic disease as a cause Check anti-centromere, anti-Scl-70, anti-U1RNP (to rule out scleroderma and other overlap syndromes)	Limited data Vascular lesions including eccentric and concentric intimal fibrosis and thrombotic lesions Venous occlusive lesions have been reported with pulmonary veins/venules Capillary congestion (101)	Phosphodiesterase-5 inhibitors Endothelin receptor antagonists Prostacyclin agonists Role for immunosuppression not clear
Pulmonary embolic disease	Usually acute onset Dyspnea Chest pain (often pleuritic) Hypoxia Occasionally hemoptysis	Thromboembolic disease usually as a result of pro-coagulant state This could include secondary antiphospholipid syndrome Severe proteinuria from lupus nephritis may result in anti-thrombin deficiency	Check aPl antibodies (LAC, aCL, anti-B2GPI) Elevated D-dimer CXR usually normal aside from potential wedge infarct CTPA	Evidence of thrombus within pulmonary arterial system	Anti-coagulation (low molecular weight heparin, oral vitamin K antagonist)
Pulmonary vasculitis	Acute dyspnea Commonly associated with fever and active extrapulmonary manifestations of SLE Hemoptysis May be initial presentation of SLE	Direct immune-mediated inflammatory response of the small vessels of the alveola resulting in increased permeability and eventually structural damage resulting in hemorrhage	CXR – bilateral alveolar interstitial infiltrates Pulmonary function tests – elevated DLCO Drop in Hb Important to check ANCA and urine dip for proteinuria/hematuria (to rule out intercurrent ANCA-associated vasculitis or pulmonary-renal syndrome)	Numerous intra-alveolar or interstitial aggregates that comprise of hemosiderin-laden macrophages Fresh hemorrhagic changes may be present in the context of DAH Capillaritis may be present (26)	IV corticosteroids Cyclophosphamide Rituximab IVIg Plasmapheresis May require mechanical ventilation
Shrinking lung syndrome	Progressive dyspnea Occasional pleuritic chest pain	Poorly understood Felt to be the result of marked diaphragmatic weakness or immobility. Possibly as a result of pleural adhesions Phrenic neuropathy also previously proposed as a possible mechanism	CXR – often non-specific, elevation of diaphragm and basal atelectasis may be seen Pulmonary function tests – restrictive pattern with reduced lung volume and DLCO	Extremely limited data from lung biopsy with features reported as alveolar microatelectasia and hyaline membrane formation (102) Post-mortem diaphragmatic tissue showing muscle atrophy (85)	Little evidence currently available to support treatment decisions Corticosteroids, Azathioprine, MMF, and Rituximab used with variable success in case reports

*CXR, chest x-ray; HRCT, high resolution computerized tomography; NSIP, non-specific interstitial pneumonia; DLCO, diffusing capacity for carbon monoxide; RhF, rheumatoid factor; CCP, cyclic citrullinated peptide; RNP, ribonuclear protein; CK, creatinine kinase; LDH, lactate dehydrogenase; aPl, antiphospholipid; IV, intravenous; MMF, mycophenolate mofetil; CT, computerized tomography; IVIg, intravenous immunoglobulin; CRP, c-reactive protein; CTPA, computerized tomography pulmonary angiogram; NSAIDs, non-steroidal anti-inflammatory drugs; ANA, anti-nuclear antibody; EKG, electrocardiogram; RVH, right ventricular hypertrophy; PASP, pulmonary artery systolic pressure; TR, tricuspid regurgitation; LAC, lupus anticoagulant; aCL, anti-cardiolipin; B2GPI, beta-2-glyoprotein-I; Hb, hemoglobin; ANCA, anti-neutrophil cytoplasm antibody; DAH, diffuse alveolar hemorrhage*.

It is important to consider that SLE-related lung disorders are likely to be under-represented due to the fact that respiratory involvement may be asymptomatic. Furthermore, serositis (pleurisy/pleural effusion) is the only respiratory symptom included in the revised 1997 American College of Rheumatology (ACR) criteria for SLE (103) and no additional respiratory manifestations were included in the 2019 combined ACR/EULAR criteria (104). In terms of measuring disease activity from pulmonary manifestations of SLE, the British Isles Lupus Assessment Group (BILAG) index includes a subsection on (cardio)respiratory features of the disease, which considers pleurisy, pleural effusion, pulmonary hemorrhage/vasculitis, interstitial lung disease, and shrinking lung syndrome as possible pulmonary manifestations of the disease (105). In comparison, the SLEDAI-2K only accounts for pleurisy as a scorable item of lupus activity involving the lungs (106). In turn, this may result in a number of patients with respiratory complications of SLE (particularly those symptoms considered more mild) to be falsely considered as either in remission or a low disease activity state (107). In comparison, the Systemic Lupus International Collaborating Clinics (SLICC)/ACR Damage Index for SLE does include a wide array of pulmonary manifestations although these are typically irreversible and thus may not be a useful measure in preventative studies (108). This has important implications for clinical trial design, which may exclude patients who have predominantly respiratory symptoms. As a result, evidence supporting therapeutic options in SLE-related lung disease are often extrapolated from other severe manifestations of the disease. Dedicated studies in the management of pulmonary disorders in SLE are greatly needed and represent a major unmet need.

## Author Contributions

RA conducted a literature review of relevant respiratory disorders. SAY, EKD, and CW expanded upon this. All authors agreed to the finalized version of this manuscript prior to submission.

## Conflict of Interest

The authors declare that the research was conducted in the absence of any commercial or financial relationships that could be construed as a potential conflict of interest.
